# Expectations about and experiences with insulin therapy contribute to diabetes treatment satisfaction in insulin-naïve patients with type 2 diabetes

**DOI:** 10.1111/j.1742-1241.2010.02363.x

**Published:** 2010-06

**Authors:** A N Naegeli, R P Hayes

**Affiliations:** Global Health Outcomes, Eli Lilly and CompanyIndianapolis, IN, USA

## Abstract

**Aim:**

The aim of this study was to investigate how patients’ expectations about and experiences with insulin therapy contribute to diabetes treatment satisfaction.

**Methods:**

The Expectations about Insulin Therapy (EAITQ) and the Experience with Insulin Therapy Questionnaires (EWITQ) were administered at baseline and end-point, respectively to insulin-naïve patients with type 2 diabetes in a randomised trial comparing treatment algorithms for inhaled insulin. Pearson correlation coefficients were calculated between EAITQ and EWITQ scores, patient characteristics and patient-reported outcomes measures. Wilcoxon Signed Rank test compared EAITQ and EWITQ item score distributions. Differences between EAITQ and EWITQ scores were calculated to categorise patients according to the extent to which their expectations were met by experiences (i.e. unmet, met, exceeded).

**Results:**

EAITQ and EWITQ data were available for 240 patients (61% male, mean age 58 years, mean diabetes duration 10 years, mean baseline HbA_1c_ 8.4%). Increasingly positive expectations were significantly associated with greater self-efficacy; greater levels of positive experiences were significantly associated with greater positive expectations, shorter diabetes duration, less symptom distress, greater well-being, self-efficacy and diabetes treatment satisfaction. Overall, patients’ experiences with inhaled insulin therapy were significantly more positive than their expectations: 58% patients’ experiences exceeded expectations, 29% patients’ experiences met expectations and 13% patients’ experiences did not meet expectations. *Post hoc* tests indicated that treatment satisfaction scores differed among these groups (all p < 0.01).

**Conclusion:**

Expectations may not independently impact treatment satisfaction, but the relationship with experiences significantly contributes to it. The EAITQ and EWITQ may be useful tools for clinicians to better understand patients’ expectations about and experiences with insulin therapy.

**What’s known**

Numerous studies have demonstrated positive and negative associations between patients’ overall satisfaction and their expectations towards and experience with products and/or services. The expectations of and subsequent experiences patients have with insulin therapy may be important determinants of treatment satisfaction, but these relationships have not been examined in the initiation of insulin therapy among insulin-naïve patients with type 2 diabetes, and to our knowledge no instruments exist to specifically assess these relationships.

**What’s new**

The Expectations about Insulin Therapy Questionnaire (EAITQ) and the Experience with Insulin Therapy Questionnaire (EWITQ) were developed to assess expectations about insulin therapy and delivery systems and experiences corresponding to those expectations, respectively. Use of these questionnaires within clinical practice may help clinicians manage patients’ treatment expectations by providing insight into the degree to which patient expectations about insulin therapy may be fulfilled through their experiences and how these factors correlate to overall treatment satisfaction.

## Introduction

Personal beliefs or perceptions about the likelihood that certain experiences will occur are referred to as expectations (1–4) Regardless of how expectations originate, their relationship with actual experiences subsequently influence an individuals’ overall satisfaction with outcomes (5–7). Numerous studies have demonstrated positive and negative associations between patients’ overall satisfaction and their expectations towards and experience with products and/or services (7–13). For example, in a sample of 344 patients, Kumar et al. (7) found that both the expectations and experiences with newly prescribed medication significantly impacted overall treatment satisfaction.

Insulin delivery options for patients with diabetes continue to expand with the development of new systems that aim to be straightforward, inconspicuous and less painful. Despite these developments, insulin-naïve patients with type 2 diabetes are still reluctant to initiate insulin therapy. Factors, or barriers, contributing to this reluctance include concerns about the treatment itself, changes to and restrictions on lifestyle, fear of taking injections, fear of hypoglycaemia, fear of weight gain and low self-efficacy pertaining to the complexity of managing insulin therapy (14–22). It is recognised that barriers may have an impact on the development of patients’ expectations (22). Instruments do exist and are commonly used by clinicians in assessing barriers to insulin therapy; however, patient-reported barriers have not previously been correlated with subsequent treatment satisfaction (14). Thus, these instruments may have limited value in predicting patient treatment satisfaction and potential adherence to insulin therapy.

The expectations of and subsequent experiences patients have with insulin therapy may be important determinants of treatment satisfaction, but these relationships have not been examined in the initiation of insulin therapy among insulin-naïve patients with type 2 diabetes, and to our knowledge no instruments exist to specifically assess these relationships. Therefore, the Experience About Insulin Therapy Questionnaire (EAITQ) and the Experience with Insulin Therapy Questionnaire (EWITQ) were developed to assess expectations about insulin therapy and delivery systems and experiences corresponding to those expectations, respectively (23).

A randomised clinical trial comparing the efficacy and safety of two treatment algorithms for an inhaled insulin provided the opportunity to administer the EAITQ and EWITQ to a sample of insulin-naïve patients with type 2 diabetes who subsequently experienced treatment with an inhaled insulin, and to address these research questions:

1. What are the correlates of expectations about and experiences with insulin therapy?
2. How do an individual patient’s expectations about insulin therapy differ from his or her experiences with insulin therapy?
3. How does diabetes treatment satisfaction differ among those individuals whose expectations about insulin therapy are exceeded by experiences with insulin therapy, those whose expectations are met by their experiences, and those whose expectations are not met by their experiences?

## Methods

This was a 6-month clinical trial conducted in 56 clinical sites in Argentina, Austria, Belgium, France, India, Mexico, Spain and United States. Primary outcomes from the study have been reported elsewhere (24). Analysis of the clinical trial primary end-points indicated that there was no significant difference between the two treatment groups. There were no placebo patients in the clinical trial. The clinical trial was conducted in agreement with the Declaration of Helsinki and the International Council on Harmonization Guidelines to good Clinical Practice. The protocol was approved by the local ethics committee or institutional review board. Participants provided informed consent prior to participation in the study.

### Study participants

Individuals with type 2 diabetes of at least 6 months duration aged 18–100 years were enrolled in the clinical trial. Patients were eligible to participate in the study if they had not achieved optimal glycemic control (A1c > 7.0% and ≤ 10.5% at screening) using two or more oral antidiabetic medications, were insulin-naïve, were non-smokers for at least 6 months prior to study start, and were able to perform pulmonary function tests per American Thoracic Society guidelines. The participants in the study reported herein were those individuals who completed the clinical trial and for whom EAITQ and EWITQ data were available both at baseline and end-point (24 weeks) (24).

### Patient-reported outcomes measures

The following self-administered Patient Reported Outcomes measures (PROs) were administered at both baseline and study end-point: Diabetes Treatment Satisfaction Questionnaire Status Version (DTSQs); Hyperglycaemia, Hypoglycaemia and Psychological subscales of the Diabetes Symptoms Checklist-Revised (DSC-R); and Well-Being Questionnaire 12 (W-BQ12). The EAITQ was administered at baseline prior to any patient education or treatment administration and the EWITQ at end-point.

The DTSQs is an eight-item instrument designed to assess satisfaction with diabetes treatment (six items) and perception of both hyperglycaemia and hypoglycaemia during the past 4 weeks. Scores for the six satisfaction items are summed for a total satisfaction score ranging from 0 to 36. Higher scores correspond to greater satisfaction. The perceived hyperglycaemia and hypoglycaemia items are analysed individually and scores range from 0 to 6, with higher scores corresponding to greater perception of unacceptable high and low blood sugars, respectively (25).

Diabetes Symptom Checklist-Revised is a 34-item instrument designed to provide a comprehensive assessment of diabetes symptom distress during the past 4 weeks (26). For this study, participants were administered only the Hyperglycaemia (four items), Hypoglycaemia (three items) and Psychological [Fatigue (four items) and Cognitive Distress (four items)] subscales. Scores range from 0 to 5 with higher symptom scores corresponding to greater symptom severity (26).

The W-BQ12 is designed to assess the well-being of patients with diabetes. It consists of three subscales (Negative Well-Being, Positive Well-Being and Energy) of four items each containing both positively worded and negatively worded items. For the Positive Well-Being and Energy subscales, higher scores correspond to a greater sense of well-being; for the Negative Well-Being subscale, higher scores correspond to a lower sense of well-being (27).

### Expectations about and experiences with insulin therapy questionnaires

Input from the U.S. Food and Drug Administration regarding the clinical trial plan for inhaled insulin necessitated the immediate development of an assessment of patients’ expectations about insulin therapy for inclusion in the randomised clinical trial. Therefore, the traditional approach for item generation (qualitative study followed by item generation and cognitive debriefing) was not possible. Item generation was instead based on quantitative and qualitative studies that have described the fears and concerns about insulin therapy of individuals with type 2 diabetes (14–22), or have reported the drivers of patient satisfaction with and preference for diabetes treatment (28). Fifteen items were developed: five items (two positively worded and three negatively worded) concerning insulin therapy in general (Table 4); five items (four positively worded and one negatively worded) concerning insulin delivery systems (Table 4); and five positively worded items to assess self-efficacy (the participant’s confidence in achieving certain outcomes using insulin therapy, such as consistently avoid high blood sugars when taking insulin).

**Table 4:** Response distributions for EAITQ and EWITQ

	EAITQ response distribution (%)			EWITQ response distribution (%)		
	DA-SDA	SIDA-SIA	A-SA	DA-SDA	SIDA-SIA	A-SA
**Taking insulin will…**						
make it easier to control my blood sugars.*	1	38	61	3	12	85
restrict my life.*	31	55	14	56	31	13
Cause me to have severe episodes of low blood sugar.*	19	72	9	50	44	7
make me feel better.*	2	49	50	5	26	68
Cause me to gain an undesirable amount of weight.	19	72	9	34	41	25
**My insulin delivery system will…**						
be physically painful.*	38	54	8	83	10	7
be easy for me to use away from home.*	5	51	45	7	20	74
not be noticed by others when I use it.	10	65	25	16	46	38
It will be easy to get the insulin dose I need with my IDS. *	2	54	44	3	14	83
My insulin delivery system will be convenient. *	4	50	46	3	18	79

* p ≤ 0.001 end-point compared with baseline. A, agree; DA, disagree; IDS, insulin delivery system; SA, strongly agree; SDA, strongly disagree; SIA, slightly agree; SIDA, slightly disagree.

The EAITQ contains the 10 items concerning insulin therapy and insulin delivery systems, all of which are preceded by ‘I expect that….’, as well as the five self-efficacy items, which are preceded by ‘I am confident that I will be able to….’ The response set for all 15 items (including the five self-efficacy items) was a 7-point scale ranging from 1 (strongly disagree) to 7 (strongly agree).

The EWITQ contains the same 10 items concerning insulin therapy and insulin delivery systems as the EAITQ, but the directions ask the respondent to refer to his or her experiences with insulin therapy in the past 4 weeks, and the items are stated in the present tense. For example, the EWITQ item, ‘Taking insulin makes me feel better’, corresponds to the EAITQ item, ‘I expect that taking insulin will make me feel better’. The EWITQ also contains the five self-efficacy questions, the wording of which is identical to that in the EAITQ.

To confirm that the 15 EAITQ items and the corresponding 15 EWITQ items covered content that was relevant to patients with type 2 diabetes, four focus groups were conducted with 27 patients receiving diabetes treatment from clinics in either Houston, Texas or Seattle, Washington. Participants who were not receiving injectable medications (*n* = 16, 62% males, 63% non-white, mean age = 50 years, mean diabetes duration = 8 years) were asked about their expectations regarding the possibility of needing to take injectable medications in the future. Participants who were currently taking injectable insulin (*n* = 11, 39% males, 45% non-white, mean age = 55 years, mean diabetes duration = 17 years) were asked how their expectations about having to take injectable medications differed from their experiences. The focus group results provided confirmation that items of the EAITQ and the EWITQ are representative of the concepts expressed by patients regarding their insulin therapy expectations and corresponding experiences, and have importance and relevance to that patient population.

A preliminary validation study was also conducted to examine factor structure, validity and reliability. This study included 294 participants (mean age = 60 years, 48% male, 67% Caucasian) from the US. The EAITQ and EWITQ demonstrated good internal reliability (Cronbach α = 0.82 and 0.79, respectively) as well as convergent and discriminant validity.

For the purposes of this study, the self-efficacy questions in the EAITQ and EWITQ were analysed as a separate subscale (Self-Efficacy subscale) with Cronbach’s alpha = 0.87 at baseline and 0.88 at end-point. For the other 10 items of the EAITQ and EWITQ, a single measure of expectations and experiences was desired. Therefore, the negatively worded items on the two questionnaires were reverse-scored so that higher scores on both positively worded and negatively worded items corresponded to more positive expectations or experiences. Internal consistency coefficients (Cronbach’s alpha) for the 10 expectations and corresponding experience items were 0.80 and 0.72, respectively.

### Statistical analyses

To test the differences between baseline values and end-point values, t-tests for dependent samples were performed. To address Research Question 1 regarding correlates of expectations about insulin therapy, Pearson correlation coefficients were calculated between EAITQ scores, patient characteristics [age, duration of diabetes, gender, body mass index (BMI)], baseline PRO scores (DTSQs, DSC-R subscales, W-BQ12 subscales and Self-Efficacy subscale) and baseline A1c. To identify the correlates of experiences with insulin therapy, Pearson correlation coefficients were also calculated between EWITQ scores, patient characteristics, end-point PRO scores, end-point A1c and EAITQ scores.

To address Research Question 2 regarding the comparison of expectations about and experiences with insulin therapy, the Wilcoxon Signed Rank test for differences in dependent samples was performed for all 10 expectation items and the corresponding experience items. This non-parametric approach was utilised because comparisons were between variables with three discrete levels.

To address Research Question 3 concerning the extent to which expectations about insulin therapy were met by experiences with insulin therapy, the EAITQ score was first subtracted from the EWITQ score to yield a difference score. Secondly, the SEM for the difference score was calculated by multiplying the standard deviation for the difference score by the square root of 1-reliability of the difference score. Difference scores were then used to categorise how expectations were met by experiences. If the difference score was zero, plus or minus one SEM of difference score, expectations were met by experience. If the difference score was greater than zero plus one SEM, expectations were exceeded by experience. If the difference score was less than zero minus one SEM, expectations were not met by experience. One-way analysis of variance with Scheffe *post hoc* tests were performed to detect whether the DTSQs scores of these three groups differed. Because of the number of analyses performed, alpha was set at p < 0.01. Calculations were computed with SPSS version 17.0 software.

## Results

The study population consisted of 240 of the 303 completers of the clinical trial for whom data for both EAITQ and EWITQ were obtained. Sixty-one per cent of the study population was male, mean age was 58 years, average length of diabetes diagnosis was 10 years, and average BMI was 32 (Table 1). Forty-four per cent of participants were from U.S. clinical sites.

**Table 1:** Study population characteristics at baseline (*n* = 240)

**Characteristic**	**Mean (SD)**
Age (years)	57.8 (9.6)
Diabetes duration (years)	10.1 (7.0)
Body mass index (kg/m^2^)	31.9 (5.8)
	**Per cent of study population**
Male	61.3
Caucasian	77.9
African American	2.1
Hispanic	15.0
East Asian	1.3
West Asian	3.8
**Country of clinical site**	
Argentina	13.8
Austria	3.3
Belgium	9.6
Spain	15.0
France	2.9
India	3.8
Mexico	7.5
United States	44.2

Across the study arms, from baseline to end-point, significant (p < 0.001) decreases were seen in study participants’ mean A1c, perceived hyperglycaemia score, hyperglycaemia symptoms score and negative well-being (Table 2). Significant (p < 0.001) increases were observed in diabetes treatment satisfaction, self-efficacy and perceived hypoglycaemia score. There were no significant (p < 0.01) changes from baseline in positive well-being or energy (Table 2).

**Table 2:** Baseline and end-point A1c and patient-reported outcomes scores (*n* = 240)

	*N*	Baseline mean (SD)	End-point mean (SD)
A1c (%)	236	8.4 (1.0)	6.9 (0.8)*
**DTSQs subscale (score minimum, maximum)**			
Satisfaction (0,36)	237	25.5 (7.3)	30.9 (5.5)*
Perceived hyperglycaemia† (0,6)	240	3.9 (1.7)	2.0 (1.6)*
Perceived hypoglycaemia† (0,6)	240	1.1 (1.6)	1.7 (1.5)*
**DSC-R subscales**			
Hypoglycaemia† (0,5)	237	1.3 (1.1)	1.1 (1.1)
Hyperglycaemia† (0,5)	238	1.5 (1.1)	1.2 (1.1)*
Psychological† (0,5)	238	1.5 (1.0)	1.3 (1.0)*
**W-BQ subscales**			
Negative well-being† (0,12)	238	2.1 (2.5)	1.5 (2.0)*
Energy (0,12)	235	7.4 (2.4)	7.6 (2.5)
Positive well-being (0,12)	237	8.4 (2.7)	8.5 (2.8)
EAITQ, EWITQ self-efficacy subscale (1,7)	239	5.5 (1.0)	5.7 (1.0)*

* p < 0.01 compared with baseline;

† lower scores represent less perceived hyperglycaemia or hypoglycaemia, negative well-being, or symptom burden. DSC-R, diabetes symptoms checklist-revised; DTSQ, Diabetes Treatment Satisfaction Questionnaire Status Version; EAITQ, Expectations About Insulin Therapy Questionnaire; EWITQ, Experience With Insulin Therapy Questionnaires; W-BQ, Well-Being Questionnaire.

### Research Question 1: What are the correlates of expectations about and experiences with insulin therapy?

Table 3 presents correlations between EAITQ scores and baseline patient characteristics and PRO assessments, between EWITQ scores and baseline patient characteristics and end-point PRO assessments, and correlations between EAITQ and EWITQ scores are also presented. The sample size of 240 could detect a correlation between variables of ± 0.246 with 90% power (two-tailed test, type 1 error rate of 0.01). A significant relationship (p < 0.01) was detected between EAITQ scores and baseline Self-Efficacy subscale scores, with more positive expectations associated with greater self-efficacy. No other significant relationships were detected between EAITQ scores and patient characteristics or baseline PRO assessments.

**Table 3:** Correlations between EAITQ or EWITQ Scores and patient characteristics and patient-reported outcomes at baseline and end-point

	Correlation with EAITQ score	Correlation with EWITQ score
EAITQ score	–	0.33*
Age	−0.11	−0.11
Diabetes duration	−0.08	−0.20*
Body mass index	0.04	−0.10
	**Baseline**	**End-point**
		
A1c	0.04	0.03
**DTSQs subscales**		
Diabetes treatmentsatisfaction	0.11	0.53*
Perceived hyperglycaemia†	0.09	−0.23*
Perceived hypoglycaemia†	−0.10	−0.13
**DSC-R subscales**		
Hypoglycaemia†	−0.04	−0.13
Hyperglyacemia†	0.05	−0.07
Psychological†	−0.04	−0.20*
**W-BQ subscales**		
Negative well-being†	−0.13	−0.17*
Energy	0.17	0.30*
Positive well-being	0.14	0.42*
EAITQ/EWITQ self-efficacy	0.46*	0.56*

* p < 0.01;

† lower scores represent less perceived hyperglycaemia or hypoglycaemia, negative well-being, or symptom burden. DSC-R, diabetes symptoms checklist-revised; DTSQ, Diabetes Treatment Satisfaction Questionnaire Status Version; EAITQ, Expectations About Insulin Therapy; EWITQ, Experience With Insulin Therapy Questionnaires; W-BQ, Well-Being Questionnaire.

Significant relationships (p < 0.01) were found between EWITQ scores and the following patient characteristics and end-point PRO assessments: diabetes duration, with more positive experiences associated with shorter diabetes duration; DTSQs Satisfaction and DTSQs Perceived Hyperglycaemia scores, with more positive experiences associated with greater diabetes treatment satisfaction and lower perceived hyperglycaemia; DSC-R psychological subscale scores, with more positive experiences associated with less symptom distress; W-BQ12 Negative Well-Being, Energy and Positive Well-Being scores, with more positive experiences associated with greater well-being; and Self-Efficacy scores, with more positive experiences associated with greater self-efficacy and expectations. EWITQ scores were also significantly associated with EAITQ scores with correlation at r = 0.33 and r-squared of 11%. These results suggest that more positive experiences are associated with more positive expectations and anticipation.

### Research Question 2: How do the item frequency distributions of expectations differ from experiences in individuals with type 2 diabetes who are insulin-naïve and who subsequently experience an inhaled insulin?

Significant differences (p ≤ 0.001) were observed between EAITQ and EWITQ item distributions (Table 4) for all but two items, ‘Taking insulin will cause me to gain an undesirable amount of weight’, and ‘My insulin delivery system will not be noticed by others when I use it’. For the positively worded items, experiences were significantly more positive than expectations. For the negatively worded items, experiences were significantly less negative than expectations.

### Research Question 3: How does diabetes treatment satisfaction differ among those individuals whose expectations about insulin therapy are exceeded by experiences with insulin therapy when compared with those whose expectations are met or those whose expectations are not met?

**Figure 1:**
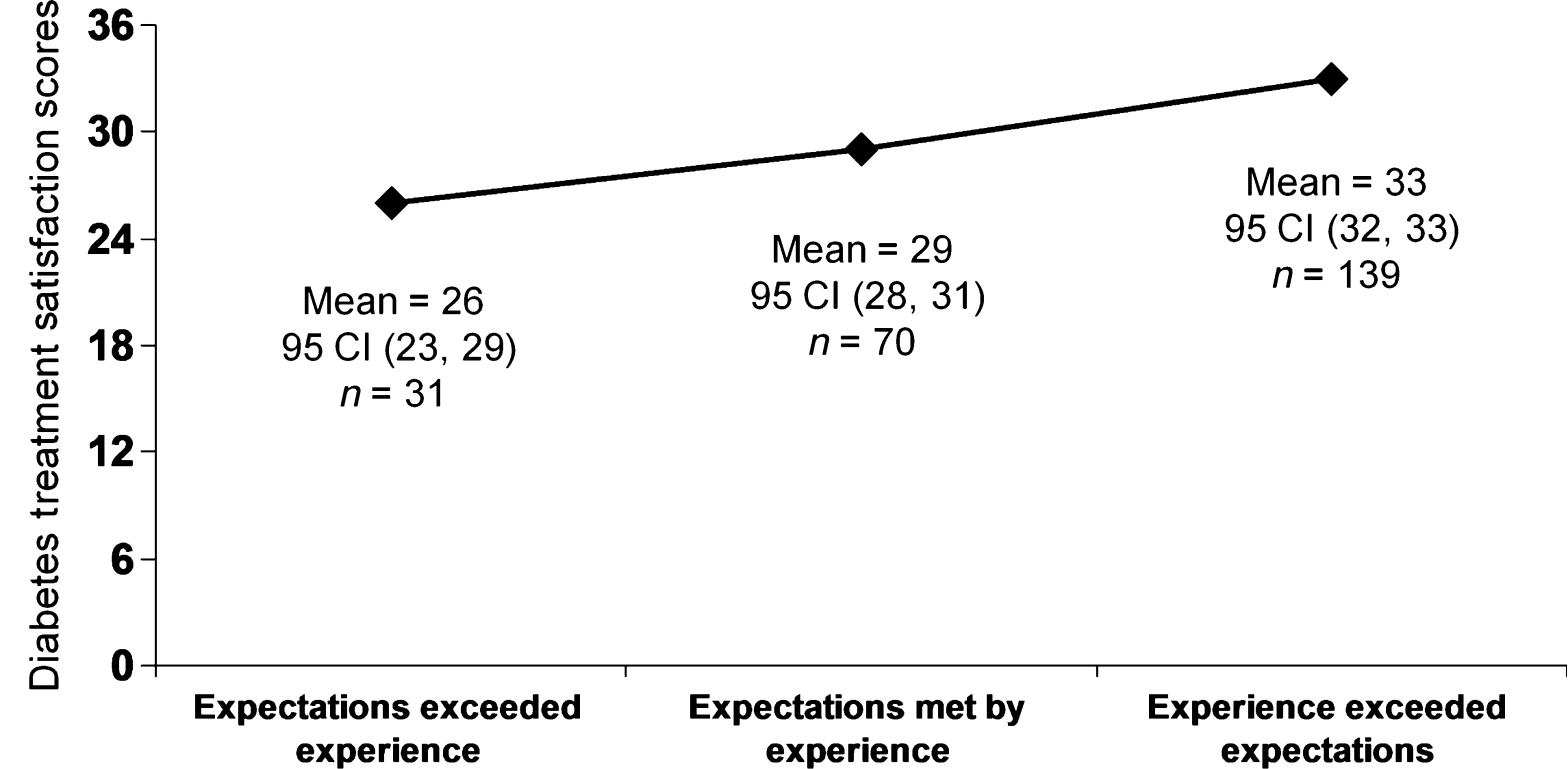
Differences in Diabetes Treatment Satisfaction Scores based on the extent to which patients’ expectations about insulin therapy are exceeded by experiences

The reliability coefficient calculated for the expectations to experience change scores was 0.69 and the standard deviation was 9.4. Therefore, the SEM for the change scores was 0.5. When change scores were calculated for each patient, 58% of patients had a change score ≥ 0.5, indicating expectations were exceeded by experience, 29% had a change score within one SEM of zero (expectations were met by experience); and 13% had change scores ≤ −0.5 (expectations were not met by experience). A significant difference in means was found between the three groups. *Post hoc* tests indicated that the mean DTSQ scores of all three groups (33, 29 and 26, respectively) were significantly different from each other (Figure 1).

## Discussion

The primary objective of this study was to identify the relationships among expectations about insulin therapy, experiences with insulin therapy, and diabetes treatment satisfaction in a sample of insulin-naïve patients with type 2 diabetes who experienced an insulin therapy. The insulin therapy was an inhaled insulin and expectations, experiences and diabetes treatment satisfaction were assessed using three validated questionnaires: EAITQ, EWITQ and DTSQ, respectively.

Expectations about insulin therapy had a significant relationship with only one of the variables explored: Self-efficacy. More positive experiences with insulin therapy were significantly associated with shorter duration of diabetes, less symptom distress, and greater well-being, self-efficacy and treatment satisfaction. The results suggest that the expectation of confidence in one’s abilities to achieve certain outcomes [e.g. avoid high blood sugars with insulin treatment (28)] may lead to more positive experiences and subsequently better treatment satisfaction. Prior research among patients with type 2 diabetes has shown that self-efficacy and outcome expectations correlate with self-care behaviours and clinical outcomes independently (4,7–13,29). However, those studies did not evaluate how these constructs correlate with overall experiences and treatment satisfaction.

Overall, patient experiences with insulin were significantly more positive than their expectations about initiating therapy, with the exception of two items: the expectation and experience of undesirable weight gain with insulin therapy, and whether the insulin delivery system would be noticeable to others. It is well recognised that patients with type 2 diabetes tend to gain weight when using insulin (15), with 16-week average gains of 0.5–6 kg for injectable (30–32) and 2.7 kg for inhaled (33) insulin. Patients in this study tended not to have any preconceived expectation about gaining weight after treatment initiation, nor did they perceive their weight gain with insulin therapy to be undesirable, even though the average change in weight was 2 kg during the study period (24). It must be recognised that patient expectations may be based on past experience with oral diabetes medications, which are typically not associated with weight gain. Additionally, the extent of weight gain that is undesirable will be based on the individual’s perception (15) and what is undesirable may only become apparent with prolonged use of insulin. Therefore, it is important for clinicians to manage patient expectations about weight gain and educate them on the ways to avoid undesirable weight gain.

There was also no significant difference between expectations and experience concerning whether patients expected and experienced the use of their insulin delivery system to be noticed by others. Discreteness of insulin delivery system has been shown to be rated as an important attribute of such systems by patients with type 2 diabetes (34) and has been suggested as a driver of insulin delivery system satisfaction and/or preference (35). Although patients may have had experience with family or friends who take insulin injections and base their expectations about the discreteness of an injection on that experience, it is unlikely that most had any experience with inhaled insulin on which to base expectations of discreteness. At the time of the study, only one inhaled insulin was on the market in the United States and it had been available for a relatively short time. No inhaled insulin was on the market in other study countries. Given that the inhaled insulin was used at mealtime, it is possible that patients found themselves in the presence of others when they needed to administer a dose. Therefore, the EWITQ item distribution may suggest that ‘others’ noticed a patient using their system, but that the attention was not a source of sufficient embarrassment or discomfort to lead a large proportion of patients to disagree that the system would ‘not be noticed by others when I use it’.

This study showed that a large proportion of patients’ expectations were exceeded by their very positive experiences with an inhaled insulin. Previous research has shown associations between symptom reduction, greater well-being and satisfaction with initiation of insulin (36,37). In the current study, statistically significant and clinically meaningful differences in diabetes treatment satisfaction were observed between three groups of patients (who did not differ in baseline characteristics) based on the relationship between their expectations and their experiences. In general, it has been shown that treatment satisfaction scores among patients with type 2 diabetes, as measured by the DTSQs, increase one to two points from baseline to end-point after treatment initiation (36,38,39). Strikingly, diabetes treatment satisfaction scores in this study increased an average of five points from baseline to end-point.

This study included approximately 20% less cases than clinical trial results as a result of the unavailability of linguistic validations of the EAITQ and EWITQ in the primary language spoken at several study sites. Results of this study are consistent with those found by Kumar (7), suggesting that comparable findings would be attained with larger sample sizes. Additional limitations to this study include that patients enrolling in a clinical trial moreover, individuals who completed the clinical trial and for whom EAITQ and EWITQ data were available both at baseline and endpoint, may not necessarily be representative of all patients with type 2 diabetes, and expectations and experiences of these patients regard an inhaled insulin delivery system and may not apply to injectable insulin delivery systems.

### Implications for diabetes educators

Using validated patient-reported outcomes measures such as the EAITQ and EWITQ in clinical research may help identify differences between expectations and experiences and determine what drives individual satisfaction or preference. Use of these questionnaires within clinical practice may help clinicians manage patients’ treatment expectations by providing insight into the degree to which patient expectations about insulin therapy may be fulfilled through their experiences and how these factors correlate to overall treatment satisfaction.

As patients begin to think about insulin therapy, it may be more advantageous for clinicians to explore patient expectations than to examine barriers to treatment initiation. Individual perceptions may be influenced through education and counselling more readily than a barrier comprised of multiple factors. Diabetes educators may use the EAITQ as a tool to assist in identifying patients with type 2 diabetes who have unrealistically positive or negative expectations about initiating treatment. These expectations can then be managed prior to initiating insulin therapy to achieve more positive experiences, better treatment satisfaction, improvements in compliance and willingness to continue treatment and, ultimately, improved outcomes.

## Clinical trials registration

This study was funded by Eli Lilly and Company and is related to clinical trial NCT00391209, ClinicalTrials.gov.

## References

[1] Kravitz RL, Callahan EJ, Paterniti D, Antonius D, Dunham M, Lewis CE (1996). Prevalence and sources of patients; Unmet expectations for care. Ann Intern Med.

[2] Kravitz RL (1996). Patients’ expectations for medical care: an expanded formulation based on review of the literature. Med Care Res Rev.

[3] Zemencuk JK, Feightner JW, Hayward RA (1998). Patients’ desires and expectations for medical care in primary care clinics. J Gen Intern Med.

[4] Miller CK, Gutschall MD, Lawrence F (2007). The development of self-efficacy and outcome expectation measures regarding glycaemic load and the nutritional management of type 2 diabetes. Public Health Nutr.

[5] Brod M, Cobden D, Lammert M, Bushnell D, Raskin P (2007). Examining correlates of treatment satisfaction for injectable insulin in type 2 diabetes: lessons learned from a clinical trial comparing biphasic and basal analogues. Health Qual Life Outcomes.

[6] Szeinbach SL, Barnes JH, Summers KH, Lenox SM (2004). Development of an instrument to assess expectations of and preference for an insulin injection pen compared with the vial and syringe. Clin Ther.

[7] Kumar RN, Kirking DM, Hass SL (2007). The association of consumer expectations, experiences and satisfaction with newly prescribed medications. Qual Life Res.

[8] Lee DS, Tu JV, Chong A (2008). Patient satisfaction and its relationship with quality and outcomes of care after acute myocardial infarction. Health Serv Outcomes Res.

[9] Lawton J, Parry O, Peel E, Douglas M (2005). Diabetes service provision: a qualitative study of newly diagnosed type 2 diabetes patients’ experiences and views. Diabet Med.

[10] Lawton J, Peel E, Parry O (2008). Patients’ perceptions and experiences of taking oral glucose-lowering agents. Diabet Med.

[11] Patrick DL, Martin ML, Bushnell DM, Pesa J (2003). Measuring satisfaction with migraine treatment: expectations, Importance, Outcomes, and Global Ratings. Clin Ther.

[12] Finch EA, Linde JA, Jeffery RW, Rothman AJ, King CM (2005). The effects of outcome expectations and satisfaction on weight loss and maintenance: correlational and experimental analyses – a randomized trial. Health Psychol.

[13] Kinmonth AL, Murphy E, Marteau T (1989). Diabetes and its care-what do patients expect?. J Rl Coll Gen Pract.

[14] Petrak F, Stridde E, Leverkus F, Crispin AA, Pfutzner A (2007). Development and validation of a new measure to evaluate psychological resistance to insulin treatment. Diabetes Care.

[15] Carver C (2006). Insulin treatment and the problem of weight gain in type 2 diabetes. Diabetes Educ.

[16] Mollema ED, Snoek FJ, Ader HJ, Heine RJ, van der Ploeg HM (2001). Insulin-treated diabetes patients with fear of self-injecting or fear of self-testing; Psychological comorbidity and general well-being. J Psychosom Res.

[17] Polonsky WH, Fisher L, Guzman S, Villa-Caballero L, Edelman SV (2005). Psychological insulin resistance in patients with type 2 diabetes. Diabetes Care.

[18] Stotland NL (2006). Overcoming psychological barriers in insulin therapy. Insulin..

[19] Funnell MM (2007). Overcoming barriers to the initiation of insulin therapy. Clin Diabetes.

[20] Hayes RP, Nakano M, Muchmore D, Schmitke J (2007). Effect of standard (self-directed) training versus intensive training for Lilly/Alkermes human insulin inhalation powder delivery system on patient-reported outcomes and patient evaluation of the system. Diabetes Technol Ther.

[21] Peyrot M, Rubin RR, Lauritzen T (2005). Resistance to insulin therapy among patients and providers. Diabetes Care.

[22] Fisher EB, Thorpe CT, DeVellis BM, DeVellis RF (2007). Healthy coping, negative emotions, and diabetes management: a systematic review and appraisal. Diabetes Educ.

[23] Hayes RP, Fitzgerald JT (2008). Perceptions, attitudes, and knowledge influence insulin delivery system satisfaction more than system used in people with type 2 diabetes.

[24] Mathieu C, Cuddihy R, Arakaki R (2009). A comparison between simplified and intensive dose-titration algorithms using AIR® Inhaled Insulin for insulin-naïve patients with type 2 diabetes in a randomized noninferiority trial. Diabetes Technol Ther.

[25] Bradley C, Bradley C (1994). The diabetes treatment satisfaction questionnaire. Handbook of Psychology and Diabetes: A Guide to Psychological Measurement in Diabetes Research and Practice.

[26] Grootenhuis PA, Snoek FJ, Heine RJ (1994). Development of a type 2 diabetes symptom checklist: a measure of symptom severity. Diabet Med.

[27] Bradley C (2000). The 12-Item Well-Being Questionnaire: origins, current stage of development, and availability. Diabetes Care.

[28] Hayes RP, Bowman L, Monahan PO (2006). Understanding diabetes medications from the perspective of patients with type 2 diabetes. Diabetes Educ.

[29] Chlebowy DO, Garvin BJ (2006). Social support, self-efficacy, and outcomes expectations: impact on self-care behaviors and glycemic control in Caucasian and African American adults with type 2 diabetes. Diabetes Educ.

[30] Duane J, Conway W (2008). Weight change in intensive insulin therapy for type 2 diabetes mellitus as a function of glycosylated hemoglobin (A1C) level achieved: the Deep South Diabetes Program. Insulin.

[31] Hermansen K, Mortensen LS, Hermansen ML (2008). Combining insulins with oral antidiabetic agents: effect on hyperglycemic control, markers of cardiovascular risk and disease. Vasc Health Risk Manage..

[32] Fajardo Montanana C, Herrero CH, Fernandez MR (2008). Less weight gain and hypoglycaemia with once-daily insulin detemir than NPH insulin in intensification of insulin therapy in overweight Type 2 diabetes patients-The PREDICTIVE BMI clinical trial. Diabet Med.

[33] Rosenstock J, Zinman B, Murphy LJ (2005). Inhaled insulin improves glycemic control when substituted for or added to oral combination therapy in type 2 diabetes: a randomized, controlled trial. Ann Intern Med.

[34] Hayes RP, Marshall TS (2006). The importance and correlates of insulin delivery system attributes in patients with type 2 diabetes. Diabet Med.

[35] Stockl K, Ory C, Vanderplas A (2007). An evaluation of patient preference for an alternative insulin delivery system compared to standard vial and syringe. Curr Med Res Opin.

[36] Wilson M, Moore MP, Lunt H (2004). Treatment satisfaction after commencement of insulin in Type 2 diabetes. Diabetes Res Clin Pract.

[37] Witthaus E, Stewart J, Bradley C (2001). Treatment satisfaction and psychological well-being with insulin glargine compared with NPH in patients with Type 1 diabetes. Diabet Med.

[38] Bradley C, Plewe G, Kliebe-Frisch C (2005). Treatment satisfaction with a basal insulin added to oral agents versus twice-daily premixed insulin alone in patients with type 2 diabetes. Diabetes.

[39] Bradley C, Speight J (2002). Patient perceptions of diabetes and diabetes therapy: assessing quality of life. Diabetes Metab Res Rev.

